# Quantitative Assessment of Clinician Assistance During Dynamic Rehabilitation Using Force Sensitive Resistors

**DOI:** 10.3389/fresc.2021.757828

**Published:** 2021-12-02

**Authors:** Margaux B. Linde, Andrew R. Thoreson, Cesar Lopez, Megan L. Gill, Daniel D. Veith, Rena F. Hale, Jonathan S. Calvert, Peter J. Grahn, Kalli J. Fautsch, Dimitry G. Sayenko, Kristin D. Zhao

**Affiliations:** ^1^Assistive and Restorative Technology Laboratory, Department of Physical Medicine and Rehabilitation, Rehabilitation Medicine Research Center, Mayo Clinic, Rochester, MN, United States; ^2^Department of Physiology and Biomedical Engineering, Mayo Clinic, Rochester, MN, United States; ^3^Mayo Clinic Graduate School of Biomedical Sciences, Mayo Clinic, Rochester, MN, United States; ^4^Department of Neurologic Surgery, Mayo Clinic, Rochester, MN, United States; ^5^Department of Neurosurgery, Center for Neuroregeneration, Houston Methodist Hospital, Houston, TX, United States

**Keywords:** spinal cord injury, paralysis, neuromodulation, force sensitive resistors, locomotor training, rehabilitation

## Abstract

**Background:** Neuromodulation using epidural electrical stimulation (EES) has shown functional restoration in humans with chronic spinal cord injury (SCI). EES during body weight supported treadmill training (BWSTT) enhanced stepping performance in clinical trial participants with paraplegia. Unfortunately, tools are lacking in availability to quantify clinician assistance during BWSTT with and without EES. Force sensitive resistors (FSRs) have previously quantified clinician assistance during static standing; however, dynamic tasks have not been addressed.

**Objective:** To determine the validity of FSRs in measurements of force and duration to quantify clinician assistance and participant progression during BWSTT with EES in participants with SCI.

**Design:** A feasibility study to determine the effectiveness of EES to restore function in individuals with SCI.

**Methods:** Two male participants with chronic SCI were enrolled in a pilot phase clinical trial. Following implantation of an EES system in the lumbosacral spinal cord, both participants underwent 12 months of BWSTT with EES. At monthly intervals, FSRs were positioned on participants' knees to quantity forces applied by clinicians to achieve appropriate mechanics of stepping during BWSTT. The FSRs were validated on the benchtop using a leg model instrumented with a multiaxial load cell as the gold standard. The outcomes included clinician-applied force duration measured by FSR sensors and changes in applied forces indicating progression over the course of rehabilitation.

**Results:** The force sensitive resistors validation revealed a proportional bias in their output. Loading required for maximal assist training exceeded the active range of the FSRs but were capable of capturing changes in clinician assist levels. The FSRs were also temporally responsive which increased utility for accurately assessing training contact time. The FSRs readings were able to capture independent stance for both participants by study end. There was minimal to no applied force bilaterally for participant 1 and unilaterally for participant 2.

**Conclusions:** Clinician assistance applied at the knees as measured through FSRs during dynamic rehabilitation and EES (both on and off) effectively detected point of contact and duration of forces; however, it lacks accuracy of magnitude assessment. The reduced contact time measured through FSRs related to increased stance duration, which objectively identified independence in stepping during EES-enabled BWSTT following SCI.

## Introduction

Spinal cord injury (SCI) affects nearly 300,000 individuals in the United States each year (1), and severe cases can result in complete, sudden paralysis below the level of injury. SCI causes a disruption in motor and sensory signals resulting in a variety of downstream secondary conditions, including urinary and bowel dysfunction, skin breakdowns, osteoporosis, and fractures (2). Currently, there are no curative treatments available to restore the functions lost due to clinically motor complete SCI. Clinical trials have been focused on the restoration of functions for individuals with SCI through neuromodulation techniques, such as epidural electrical stimulation (EES). In recent years, several case reports have demonstrated that EES paired with task specific training has enabled motor recovery of stepping function for a select number of individuals with SCI (3–5). Although motor activation below the level of injury is possible during initial use of EES, physical therapy has been critical in restoring motor function necessary for dynamic stepping. However, there are challenges in differentiating clinician assistance from participant's progression when performing rehabilitation tasks; specifically, during body weight supported treadmill training (BWSTT).

The delivery of BWSTT has included numerous approaches since its inception, but most models require four highly skilled clinicians experienced in providing tactile cues and support for individuals with SCI. Detailed recovery models, designed to progressively regain lost functions, are an alternative option to current guidelines of standard-of-care therapy. The BWSTT principles instruct clinicians on optimal hands-on support at the legs, progressively increasing limb loading and repetition of steps for maximal sensory input (6). The rehabilitation techniques applied during BWSTT, including adjustments to both ascending and descending inputs, such as body weight support (BWS), speed, or verbal cueing, can enhance or diminish functional output affecting the need for clinician assistance (7).

The tools to measure independence and progression of functional recovery with the use of EES are imperative to provide support for continued therapeutic care and are necessary to demonstrate progression of function. Other forms of stimulation, such as non-invasive transcutaneous stimulation, have shown improvement in independence through measurement of interactive applied forces with force sensitive resistors (FSRs). The FSRs are commercially available and manufactured from thin, flexible polymers that allow easy adaptation to locations where forces may be applied. The internal resistance of the FSR changes when force is applied to the active area of the sensor, which affects the output voltage and current passing through it (8). The FSRs have been used in a variety of biomechanical environments and have been shown to be useful for measuring loads between soft tissue and devices such as prosthetics (9). For example, Sayenko et al. (10) conducted a study where FSRs captured reduced applied forces at the knees during stimulation-enabled static standing activities, in correlating with leg extension activation. Additionally, the FSRs have been used in studies to assess applied forces at the waist when assistance was required for stabilization for individuals who have suffered a stroke (11), and they are routinely used in biomechanical studies involving insole sensors applied during gait analysis to detect stance and swing phase (12).

The studies that report functional return with the use of EES and rehabilitation interventions have yet to classify clinician assistance using objective measures during BWSTT to quantify performance progress. In this study, the FSRs were incorporated during dynamic BWSTT stepping assessments, with the sensors overlying the tibial tuberosity of participants to assess applied forces during the stance phase of the gait cycle to monitor progression toward maximal independence and minimal clinician assistance. Therefore, the goal of this study was to validate the FSRs for application during dynamic stepping activities, and to verify timing and force application of clinician assistance, which may indicate participant progression toward independence during BWSTT.

## Materials and Methods

### Validation

**Figure 1:**
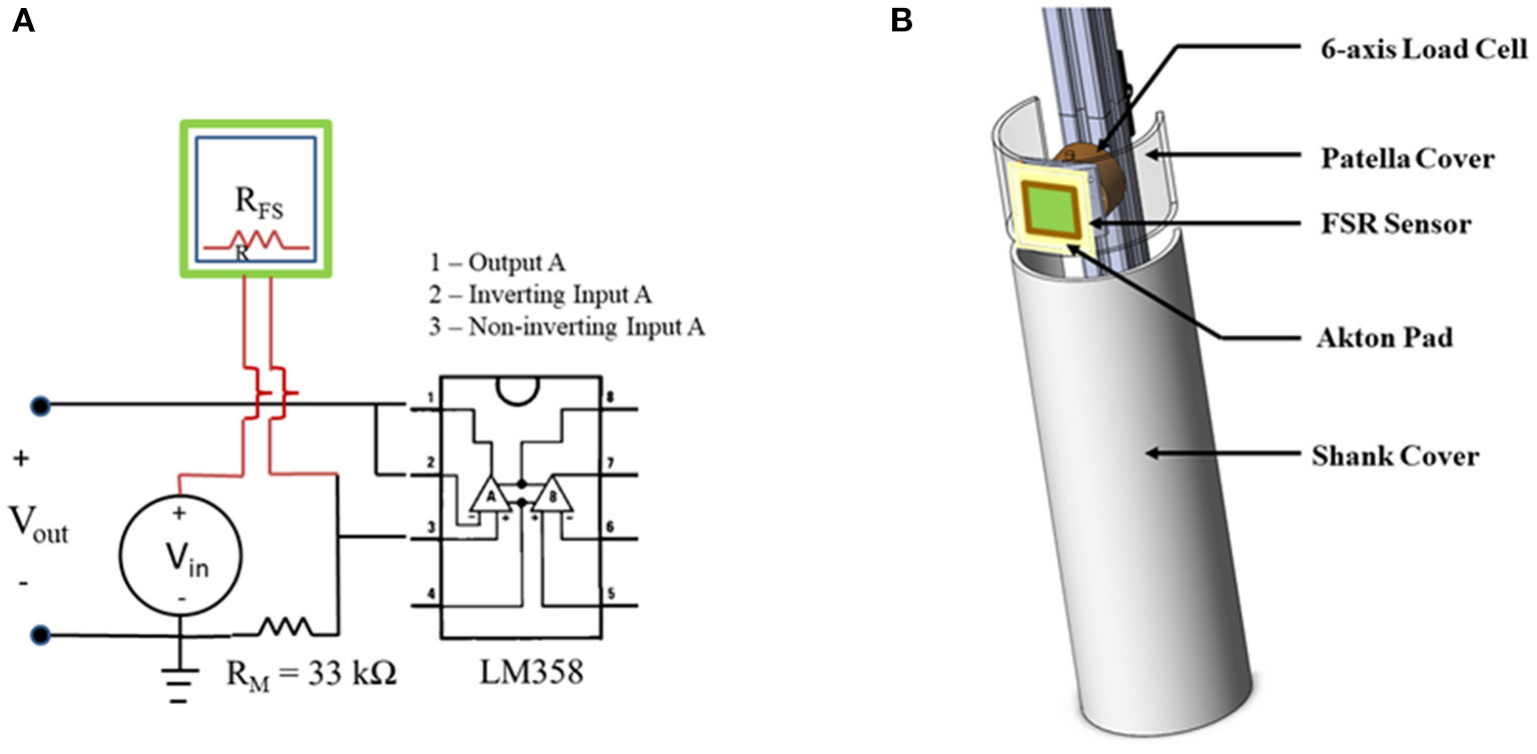
Validation set up. **(A)** Diagram of force sensitive resistors (FSR) sensor electrical circuit with amplification. **(B)** 3D sketch of design of instrumented lower leg (ILL) used for FSR validation testing.

Square FSR sensors (Model 406, Interlink Electronics, Irvine, CA) were soldered to 10-foot leads with a Bayonet Neill-Concelman (BNC) connector on the terminal end. The FSRs were connected to an operational amplifier circuit powered with a direct current (DC) supply voltage of 5 V (Figure 1A). Before use, the FSRs were calibrated by laying the sensors on a flat, hard surface, placing cylindrical masses ranging from 200 to 5,000 g to the center of the sensor padded with a 2-cm-square piece of felt. After performing a logarithmic transformation, a linear equation was fit to the data using least squares linear regression to determine the applied load /output voltage relationship using coefficients c_1_ and c_0_ (linear slope and y-intercept, respectively) (Equation 1).


(1) $$\begin{array}{l} FSR\;Force\;\left(in\;Newtons\right)=10ˆ\;\left(c_{1}\cdot \;Voltage+c_{0}\right)\cdot \;0.00981 \end{array}$$

An instrumented lower leg model (ILL) was constructed from polyvinyl chloride (PVC) tubing (5-inch outer diameter), extruded structural aluminum, metal and polymer hardware, and a six-axis load cell (Mini58, ATI Industrial Automation, Apex, NC) to document applied forces to the leg to serve as a gold standard (Figure 1B). The ILL included two components, an active patellar region, ~3.5 inches long into which the load cell was installed and a mechanically isolated tibia region. To simulate soft tissue at the knee, an Akton action pad (Action Products Inc., Hagerstown, MD) was attached on the patellar surface. The ILL was mounted to the framework of a treadmill that was customized for locomotor training. An FSR was calibrated and applied to the region simulating the tibial tuberosity, over the Akton pad, using double-sided tape.

Two clinicians, experienced in locomotor training, participated in this validation study. The clinicians applied load to the patella surface of the ILL to simulate contact with a recipient of locomotor training. Each clinician applied loads to the ILL at three different load levels (low, medium, and high) that were based on their tactile feel for the range of loading applied clinically. Sets of 10 repeated loads (each representing a leg step) were applied at each load level with short breaks between each set. To drive a frequency of load application that would be consistent and similar to real locomotor training conditions, clinicians applied loads in cadence with a metronome set at a rate of 70 beats per minute. Both clinicians applied loading sets to the ILL positioned on the framework to represent a recipient's right-side leg; the ILL was then repositioned to represent a recipient's left-side leg. Each clinician applied a total of 60 loads to the ILL. The FSR and ILL loads were recorded at a sample rate of 4 kHz (Powerlab 35/16, AD Instruments, Colorado Springs, CO).

### Participants

Two male participants, both diagnosed with traumatic, sensorimotor complete SCI and American Spinal Injury Association (ASIA) Impairment Scale Grade A (AIS-A) (13) were enrolled in a feasibility clinical trial for EES (National Clinical Trial Number: 02592668). Participant 1 began the trial at age 26, with injury level located at the level of T6, SCI occurred 3 years prior. Participant 2 began the trial at age 37, injury level located at the level of T3, SCI occurred 6 years prior. Each participant provided written informed consent to all procedures approved under the Mayo Clinic Institutional Review Board (IRB 15-000510) with US Food and Drug Administration Investigational Device Exemption (IDE G150167).

### Protocol

The participants completed 6 months of locomotor training following protocols laid out by Locomotor Training Principles and Practice (6). At the end of the 6 months, both participants were implanted with the Medtronic SureScan Ultra, 5-6-5 epidural array (Medtronic, Minneapolis, MN, USA), followed by 1 month of recovery. When the participants returned to the laboratory, stimulation was initiated, and parameters were adjusted for optimal lower extremity movement over the next several months (3). Following each month of BWSTT, the participants engaged in a data collection session within the BWS treadmill system (Power NeuroRecovery Inc., Louisville, KY, USA). The participants were suspended over the treadmill with a climbing harness (Robertson Harness, Ft. Collins, CO, USA), and BWS was provided for appropriate loading and step production for both clinicians and participants to achieve optimal stepping patterns. Four clinicians assisted in step training, one at each leg, one at the hips, and one running the computerized treadmill and BWS system. Data acquisition software (Labchart, ADInstruments, Colorado Springs, CO, USA) was used to synchronize data from in-shoe sensors (F-Scan System, Tekscan, South Boston, MA, USA) to identify stance phase of the gait cycle and FSRs, which were positioned bilaterally at the tibial tuberosity of the participant for detection of clinician assistance during BWSTT. The locomotor training principles described above were used for appropriate facilitation of leg movements, and clinicians had been expertly trained to apply tactile cueing at the knees for appropriate leg activation and joint manipulation throughout the step cycle. Data collection timepoints for “no EES” and “EES on” conditions occurred at months 6, 12, and 18, where month 6 corresponds to the pre-implant period.

### Clinician Assistance and Progression (Independence)

Optimal parameters were systematically determined through testing of active electrode configurations and adjustments in stimulation parameters that have been described in our previous publication (14). These EES parameters were utilized during “EES on,” and treadmill speeds and BWS settings were optimized prior to each treadmill testing session. The clinician-applied forces were measured during each trial. The participants were instructed to perform intentional active stepping during BWSTT testing with “EES on” and “no EES.” The participants were tasked with determining what conditions (stimulation parameters, BWS, and treadmill speed) would enable the most independence during stepping. Both the participants preferred lower BWS (0–30%), and self-selected, slower treadmill speed (0.5–0.8 mph), and using parallel bars for arm support enabling trunk control during weight shifting. The participants were encouraged to avoid loading weight through their arms. In this study, we describe independence as requiring minimal to no clinician assistance at the anterior knee (tibial tuberosity) during the stance phase of the gait cycle. For simplicity purposes, we do not include independence for the swing phase of the gait cycle.

## Data Analysis

### FSR Validation

The steps for which the FSR load on the ILL did not exceed the measurement range were identified for analysis. Peak FSR load and peak resultant applied force on the load cell were calculated for these steps. The duration of contact was also assessed for all steps. Bland-Altman plots were generated for applied force and duration data to identify the presence of any offset or proportional biases in the FSR response. Offset biases were tested using a Wilcoxon signed rank test (significance level *p* < 0.05). Pearson Correlation Coefficients were calculated, and straight lines were fit to the data using linear regression to characterize any proportional bias.

### Data Processing

Each participant dataset consisted of ~10 gait cycles. Up to four trials were used for each timepoint. The data was then segmented and processed in a custom-made MATLAB algorithm (MATLAB 2019a, The MathWorks Inc., Natick, MA, USA). Each gait cycle duration was identified by Tekscan in-shoe sensors. The stance and swing phase of the gait cycle were identified similar to our previous publication (7). The duration of the applied force was identified through FSRs and a minimum threshold of 5 N determined initiation and termination of assistance. The maximum load was calculated based on the highest value within the duration of the applied force. The median and range were calculated for each trial, condition, and timepoint. Clinician assistance and progression assessed through stance and applied force duration were calculated independently for participant 1 and participant 2. Conditions for each test session include “no EES” and “EES on” where participants were instructed to use active intentional treadmill stepping with low BWS and self-selected slower speed.

## Results

### Validation Data

Of the 120 loads applied to the ILL by the two clinicians who participated in the validation study, 52 attempts resulted in loads that did not exceed the capacity of the FSR sensor. For the low-level loading, 40 of 40 loads registered on the FSR (20 per clinician), 12 of 40 medium-level loads registered (5 for clinician 1 and 7 for clinician 2), and 0 of 40 high-level loads registered. The gold standard load cell indicated that the median (range) of low-level applied force by the clinicians was 61.8 N (29.1–102.9 N), the median (range) of medium-level applied force was 124.7 N (82.3–261.0 N), and the median (range) of high-level applied force was 304.7 N (230.9–385.5 N).

**Figure 2:**
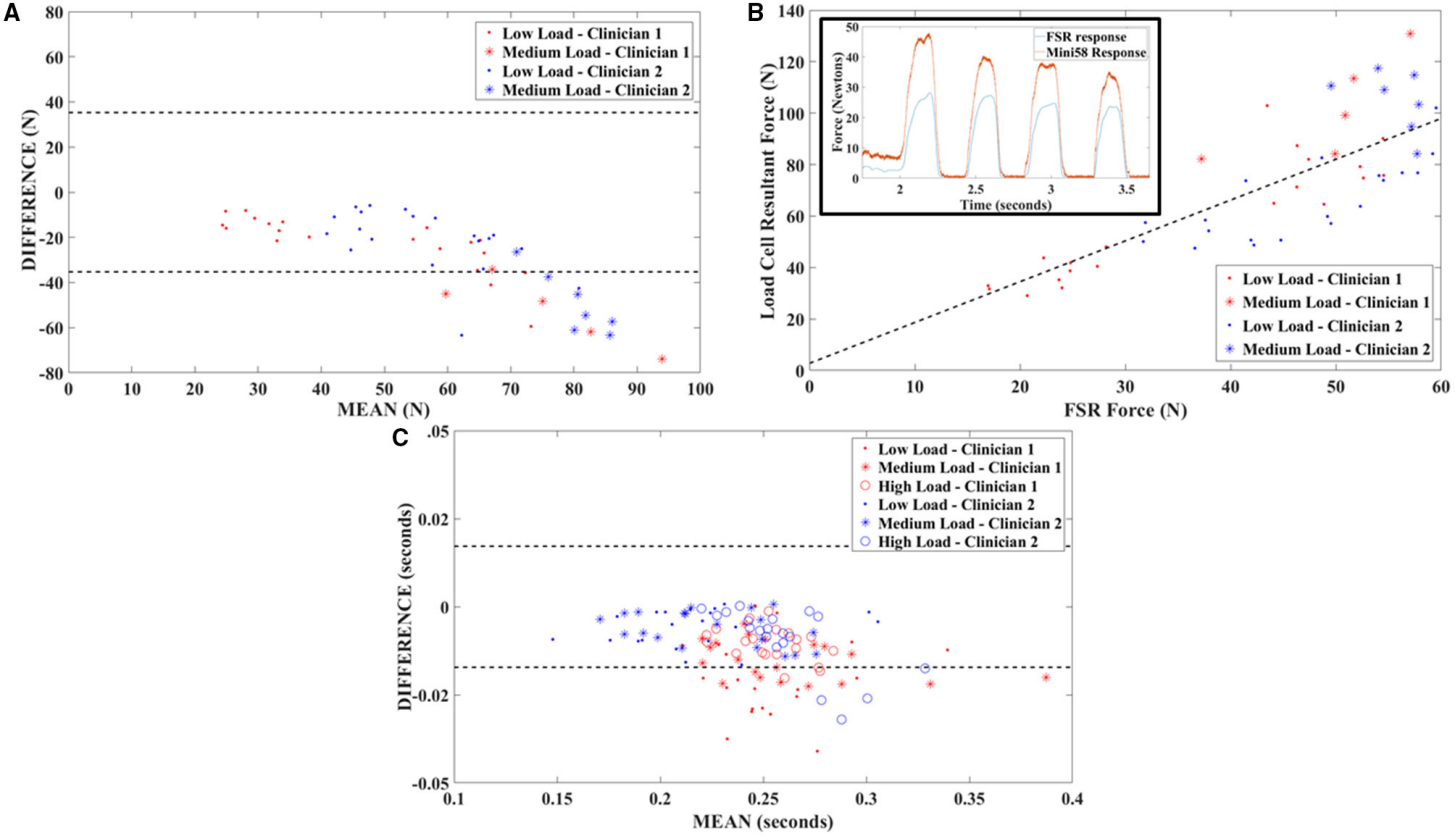
Validation testing outcomes. **(A)** Bland-Altman plot of FSR validation testing data showing a proportional bias between FSR output and gold standard (six-axis load cell) force measurements. Dashed lines indicate ±1.96 SDs. **(B)** Correlation plot for FSR and gold standard output with best fit line obtained through least squares linear regression. The insert depicts time series data and illustrates responsiveness of the FSR sensors. **(C)** Bland-Altman plot of FSR validation testing data showing a small offset bias between FSR output and gold standard (six-axis load cell) load duration measurements. Dashed lines indicate ±1.96 SDs.

The Bland-Altman plot for applied force results indicated the presence of an offset bias between the response of the FSR and that of the gold standard; the FSR response magnitude consistently falls below that of the gold standard, and the bias was confirmed by the Wilcoxon signed rank test (*p* < 0.001; Figure 2A). The trend on the Bland-Altman plot also indicated a proportional bias in the FSR response. The correlation coefficient between FSR and load cell applied force was 0.77. The slope and intercept of the best fit regression line were 1.58 and 2.80, respectively, with a coefficient of determination of 0.60 (Figure 2B). The Bland-Altman plot for duration of applied load indicated the presence of an offset bias with FSR duration consistently falling below that obtained from the gold standard load cell, confirmed by the Wilcoxon signed rank test (*p* < 0.001; Figure 2C). The mean bias was quite a small (0.011 s) and bias appears to be independent of maximum load applied.

### Participant Data

**Figure 3:**
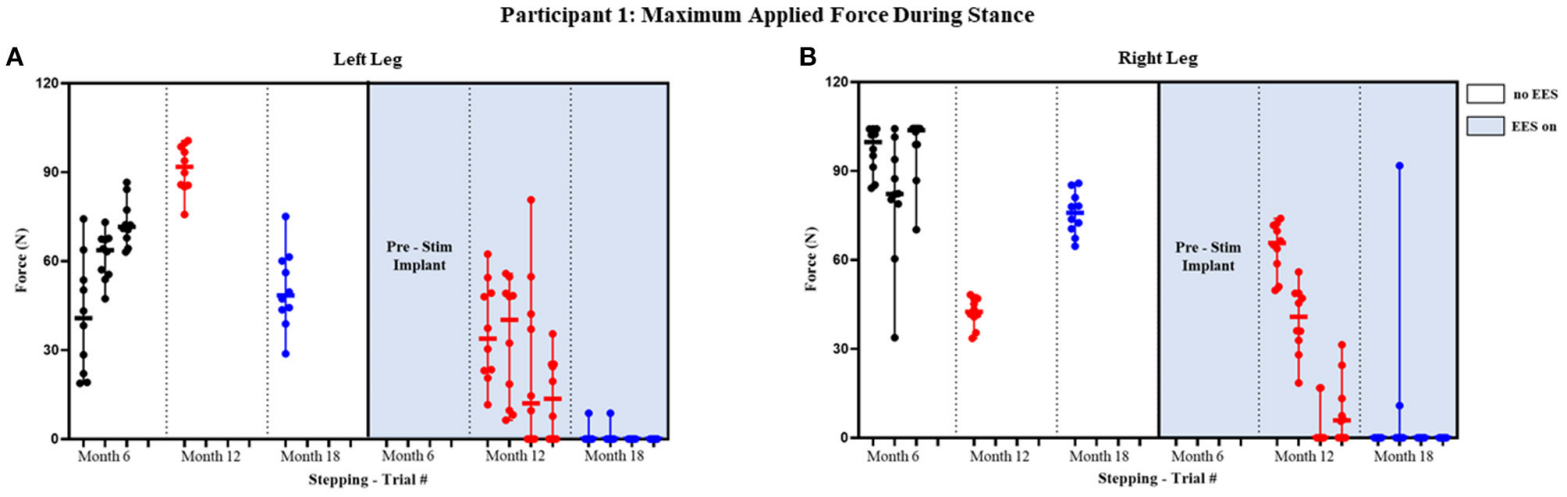
Participant 1: maximum applied force during stance. Maximum applied force during stance phase of the gait cycle with line at the median and range for left leg **(A)** and right leg **(B)**. Each tick in the x-axis represents a trial of up to 10 consecutive stance cycles. The dots represent the maximum force for each consecutive cycle where black, red, and blue dots correspond to month 6, 12, and 18, respectively.

**Figure 4:**
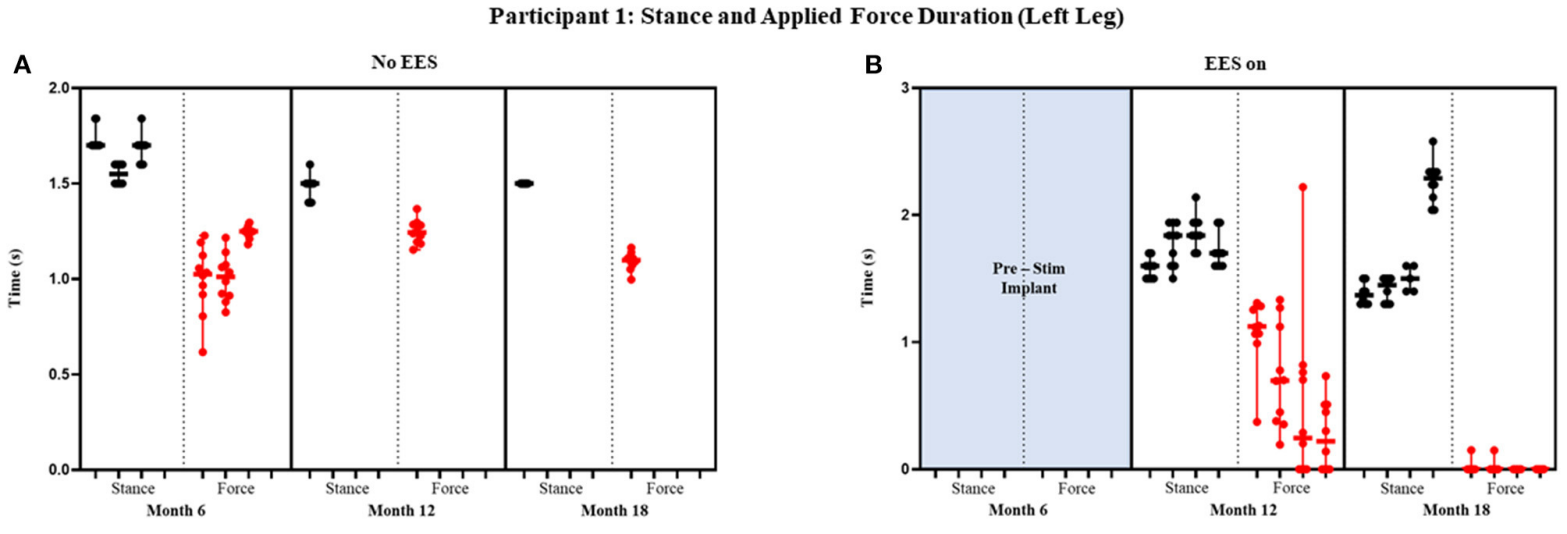
Participant 1: stance and applied force duration left leg. Duration of stance (black dot) and applied force (red dot) with line at the median and range for left leg during no EES **(A)** and EES on **(B)**. Each tick in the x-axis represents a dataset of up to 10 consecutive cycles.

**Figure 5:**
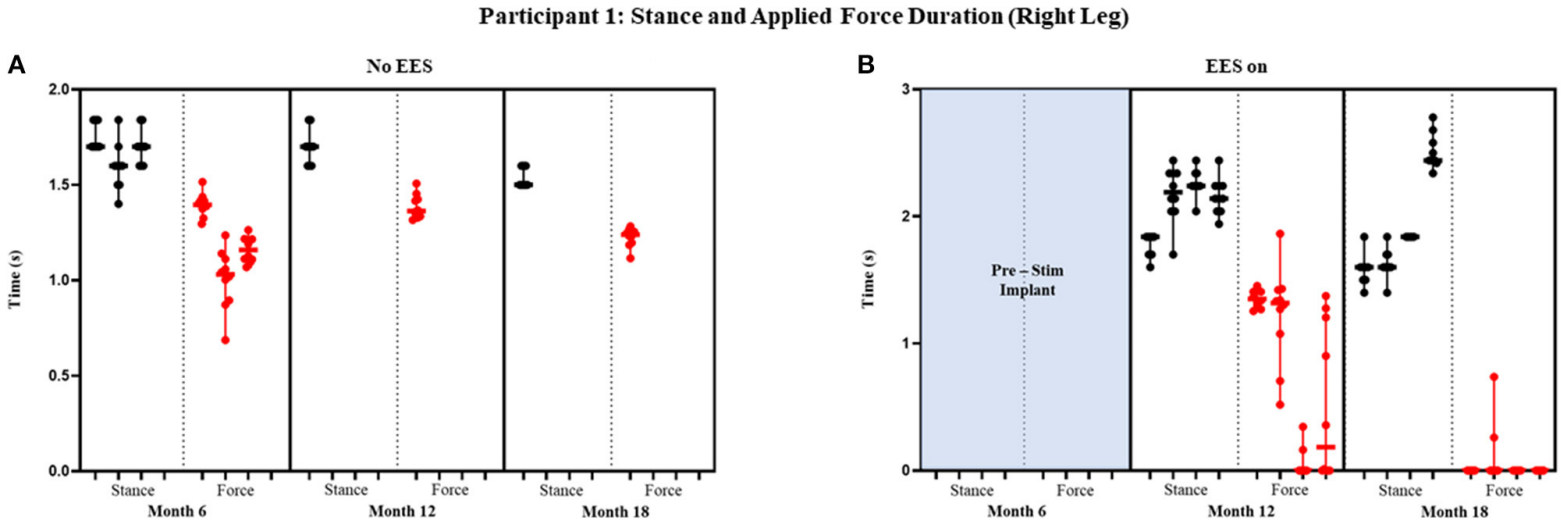
Participant 1: stance and applied force duration right leg. Duration of stance (black dot) and applied force (red dot) with line at the median and range for right leg during no EES **(A)** and EES on **(B)**. Each tick in the x-axis represents a dataset of up to 10 consecutive cycles.

**Figure 6:**
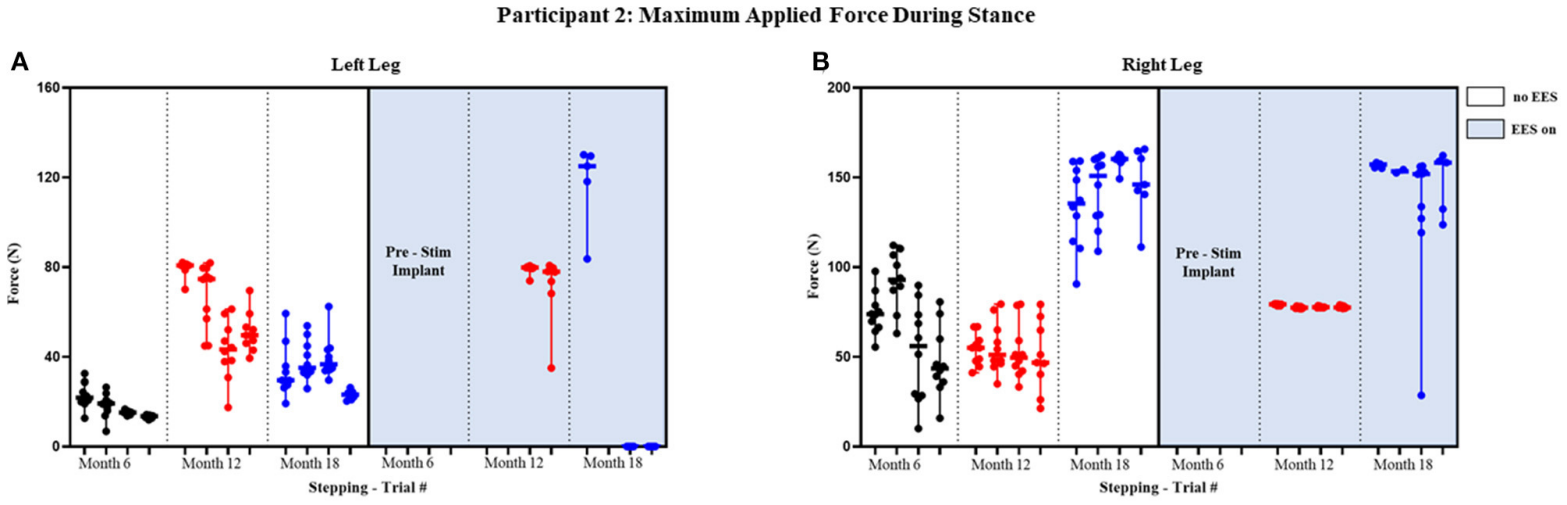
Participant 2: maximum applied force during stance. Maximum applied force during stance phase of the gait cycle with line at the median and range for left leg **(A)** and right leg **(B)**. Each tick in the x-axis represents a trial of up to 10 consecutive stance cycles. The dots represent the maximum force for each consecutive cycle where black, red, and blue dots correspond to month 6, 12, and 18, respectively.

**Figure 7:**
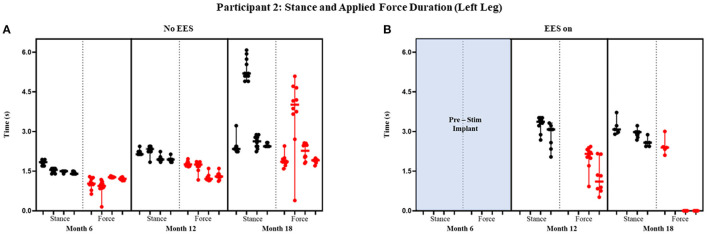
Participant 2: stance and applied force duration left leg. Duration of stance (black dot) and applied force (red dot) with line at the median and range for left leg during no EES **(A)** and EES on **(B)**. Each tick in the x-axis represents a dataset of up to 10 consecutive cycles.

**Figure 8:**
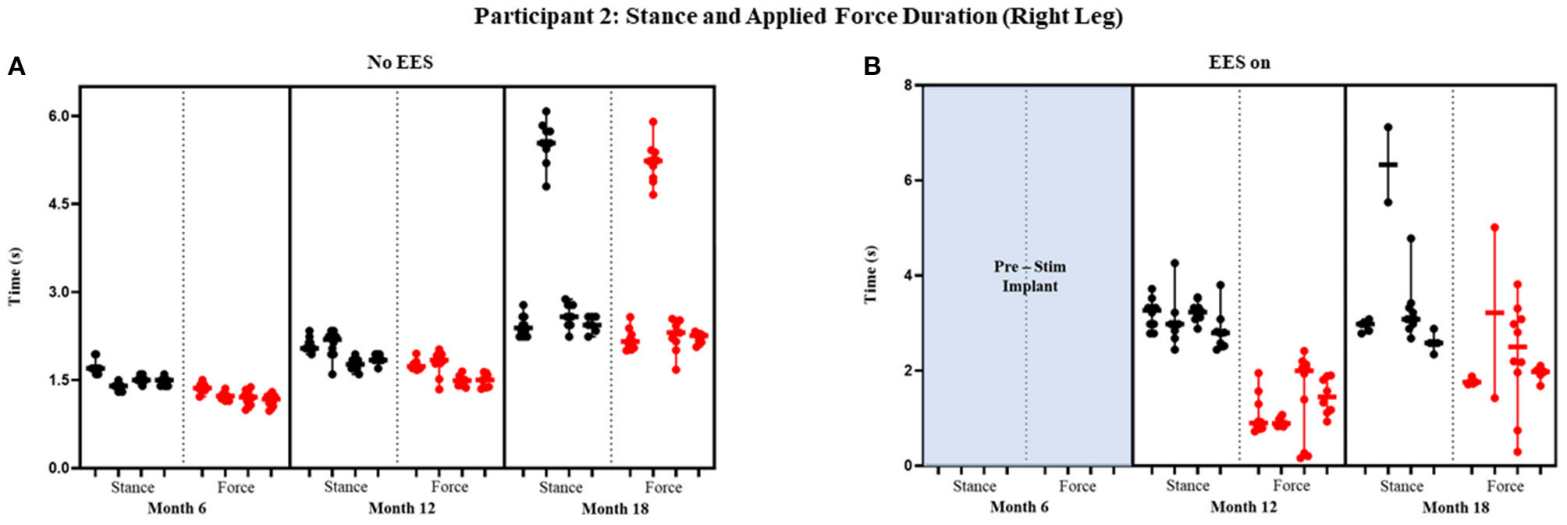
Participant 2: stance and applied force duration right leg. Duration of stance (black dot) and applied force (red dot) with line at the median and range for right leg during no EES **(A)** and EES on **(B)**. Each tick in the x-axis represents a dataset of up to 10 consecutive cycles.

Participant 1 data including the median range of the maximum applied forces (*N*) for both legs at each timepoint during the stance phase of the gait cycle are presented in Figure 3, Figures 4 and 5 show the variability of the duration of stance and applied force (seconds) during the stance phase for the left leg and right leg. Likewise, participant 2 data showing maximum applied forces (median and range) for both legs at each timepoint during the stance phase of the gait cycle are presented in Figure 6, wherein, Figures 7 and 8 show the variability of the duration of stance and applied force (seconds) during the stance phase for the left leg and right leg. The unshaded (half-left side) and shaded (half-right side) areas of each plot represent the datasets with “no EES” and “EES on,” respectively. These dot plots show the variability of the applied force of each stance phase during each trial. Similarly, due to the complexity and dynamics of the study, some timepoints do not have four trials. Participant safety played a large role during testing, ensuring each was able to continue with the task of BWSTT before adding more trials. Inconsistencies in the number of trials collected are also due to the intensive nature of BWSTT, and at times, the integrity of the FSRs was compromised potentially from mismanagement of wires or damage to the sensors. Participant 2 had more trials than participant 1; however, there were more errors present in testing overall, which are noted as not available (NA) in **Table 2**.

### Participant 1

#### Peak Forces Stance Phase

The median peak applied forces reported between FSRs (right and left) and between timepoints are highly variable. During stance phase with “no EES” condition, force was applied for all steps within all trials indicating the need for assistance during knee extension. Overall, the median applied forces for the left leg, during “no EES,” were always higher than “EES on.” However, the right leg had a single trial during month 12 where the median applied force was higher during “EES on” (Table 1A). The overall applied force range for both legs during “no EES” was between 18.79 and 104.46 N, while “EES on” had an overall range of 0–91.79 N. The decrease in the applied median force range, during “EES on,” for both legs, suggests the level of clinician assistance declined with time. In other words, minimal applied force was necessary during the stance phase (Figure 3).

**Table 1:** Participant 1: (A) maximum applied force values for left and right leg including all trials at month 6, 12, and 18 and (B) stance and applied force duration values for left and right legs including all trials at month 6, 12, and 18.

**PARTICIPANT 1**												
**(A)**	**Left leg max applied force (** ***N*** **)**						**Right leg max applied force (** ***N*** **)**					
	**No EES**			**EES on**			**No EES**			**EES on**		
**6 month**	**Trial**	**Median**	**Range**	**Trial**	**Median**	**Range**	**Trial**	**Median**	**Range**	**Trial**	**Median**	**Range**
	1	40.73	18.79–86.58	Pre-implant			1	99.79	33.80–104.36	Pre-implant		
	2	63.71					2	82.25				
	3	71.70					3	103.8				
**12 month**	**Trial**	**Median**	**Range**	**Trial**	**Median**	**Range**	**Trial**	**Median**	**Range**	**Trial**	**Median**	**Range**
	1	91.85	71.72–100.62	1	33.86	0.0–80.68	1	42.48	33.57–48.26	1	65.72	0.0–74.01
				2	40.19					2	40.79	
				3	12.07					3	0.00	
				4	13.57					4	5.88	
**18 month**	**Trial**	**Median**	**Range**	**Trial**	**Median**	**Range**	**Trial**	**Median**	**Range**	**Trial**	**Median**	**Range**
	1	48.46	28.72–75.08	1	0.00	0.0–8.72	1	75.87	64.64–85.86	1	0.00	0.0–91.79
**(B)**	**Left leg stance and applied force duration (s)**						**Right leg stance and applied force duration (s)**					
	**No EES**			**EES on**			**No EES**			**EES on**		
**6 month**	**Trial**	**Stance**	**Applied force**	**Trial**	**Stance**	**Applied force**	**Trial**	**Stance**	**Applied force**	**Trial**	**Stance**	**Applied force**
	1	1.70	1.02	Pre-implant			1	1.70	1.40	Pre-implant		
	2	1.55	1.01				2	1.60	1.03			
	3	1.70	1.25				3	1.70	1.16			
**12 month**	**Trial**	**Stance**	**Applied force**	**Trial**	**Stance**	**Applied force**	**Trial**	**Stance**	**Applied force**	**Trial**	**Stance**	**Applied force**
	1	1.50	1.24	1	1.60	1.12	1	1.70	1.37	1	1.84	1.35
				2	1.84	0.70				2	2.19	1.32
				3	1.84	0.25				3	2.24	0.00
				4	1.70	0.22				4	2.14	0.18
**18 month**	**Trial**	**Stance**	**Applied force**	**Trial**	**Stance**	**Applied force**	**Trial**	**Stance**	**Applied force**	**Trial**	**Stance**	**Applied force**
	1	1.50	1.10	1	1.32	0.00	1	1.50	1.24	1	1.55	0.00
				2	1.45	0.00				2	1.60	0.00
				3	0.70	0.00				3	0.92	0.00
				4	2.29	0.00				4	2.44	0.00

#### Stance and Applied Force Duration

Figures 4 and 5 show the duration of the applied force and stance duration for each timepoint during “no EES” and “EES on” for each leg. Conditions with “no EES” and “EES on” demonstrated that the applied force duration did not exceed the stance phase for both legs. This suggests that the clinicians did not have to apply force for the whole duration of stance. The stance duration during “EES on” was consistently greater than stance with “no EES.” Compared with “no EES,” steps taken with “EES on” required the least duration of applied force on the right leg at 0 s and only slightly higher on the left leg ranging from 0.22 to 1.12 s at most. The applied force for nearly all trials at month 18 fell below 1 s with many at 0 s, indicating progression toward independence in stance phase (Table 1B).

### Participant 2

#### Peak Applied Force Stance Phase

The median peak applied forces reported between FSRs (right and left) and between timepoints are highly variable. Overall, the median peak applied forces were higher during “EES on” for the left leg. The right leg, on the other hand, had similar applied forces for both conditions (Figure 6). During month 12, two trials had dropped FSR data that were indicated by NA. During month 18, for “ESS on,” the left leg had some trials that reported an applied force of 0 N indicating some steps unilaterally required no assistance during the stance phase. The right leg always required applied load for both conditions, where the lowest applied force (10.05 N) occurred during “No EES” (Table 2A).

**Table 2:** Participant 2: (A) maximum applied force values for left and right leg including all trials at month 6, 12, and 18 and (B) stance and applied force duration values for left and right legs including all trials at month 6, 12, and 18.

**PARTICIPANT 2**												
**(A)**	**Left leg max applied force (** ***N*** **)**						**Right leg max applied force (** ***N*** **)**					
	**No EES**			**EES on**			**No EES**			**EES on**		
**6 month**	**Trial**	**Median**	**Range**	**Trial**	**Median**	**Range**	**Trial**	**Median**	**Range**	**Trial**	**Median**	**Range**
	1	21.82	6.80–32.58	Pre-implant			1	73.77	10.05–112.18	Pre-implant		
	2	19.19					2	92.98				
	3	15.18					3	56.00				
	4	13.54					4	43.56				
**12 month**	**Trial**	**Median**	**Range**	**Trial**	**Median**	**Range**	**Trial**	**Median**	**Range**	**Trial**	**Median**	**Range**
	1	80.77	17.42–81.87	1	NA	Undetermined	1	55.00	21.26–79.39	1	>80^*^	Max >80^*^
	2	74.69		2	NA			51.10		2	>80^*^	
	3	43.28		3	79.83			49.51		3	>80^*^	
	4	48.47		4	75.75			46.54		4	>80^*^	
**18 month**	**Trial**	**Median**	**Range**	**Trial**	**Median**	**Range**	**Trial**	**Median**	**Range**	**Trial**	**Median**	**Range**
	1	29.56	19.21–62.45	1	41.8	0–149.41	1	135.4	90.56–165.74	1	157.3	28.48–162.17
	2	35.09			0.00			150.9			153.4	
	3	36.67			0.00			160.3			151.9	
	4	21.56			0.00			141.6			158.2	
**(B)**	**Left leg stance and applied force duration (s)**						**Right leg stance and applied force duration (s)**					
	**No EES**			**EES on**			**No EES**			**EES on**		
**6 month**	**Trial**	**Stance**	**Applied force**	**Trial**	**Stance**	**Applied force**	**Trial**	**Stance**	**Applied force**	**Trial**	**Stance**	**Applied force**
	1	1.84	1.03	Pre-implant			1	1.70	1.36	Pre-implant		
	2	1.55	0.94				2	1.40	1.23			
	3	1.50	1.27				3	1.50	1.21			
	4	1.40	1.21				4	1.50	1.17			
**12 month**	**Trial**	**Stance**	**Applied force**	**Trial**	**Stance**	**Applied force**	**Trial**	**Stance**	**Applied force**	**Trial**	**Stance**	**Applied force**
	1	2.14	1.76	1	NA	NA	1	2.04	1.74	1	3.27	0.90
	2	2.34	1.75	2	NA	NA	2	2.19	1.84	2	2.91	0.86
	3	1.94	1.21	3	3.37	2.15	3	1.77	1.50	3	3.23	2.00
	4	1.94	1.30	4	2.83	0.86	4	1.84	1.50	4	2.68	1.45
**18 month**	**Trial**	**Stance**	**Applied force**	**Trial**	**Stance**	**Applied force**	**Trial**	**Stance**	**Applied force**	**Trial**	**Stance**	**Applied force**
	1	2.34	1.85	1	1.45	1.05	1	2.39	2.16	1	3.08	1.75
	2	5.02	4.01	2	NA	NA	2	5.54	5.24	2	6.33	3.21
	3	2.63	2.27	3	2.98	0.00	3	2.58	2.3	3	3.04	2.50
	4	2.55	1.83	4	1.22	0.00	4	2.49	2.15	4	2.58	1.97

* *Indicates FSR load capacity was exceeded for all measurements*.

#### Stance and Applied Force Duration

Figures 7 and 8 show the duration of the applied force and stance for each timepoint during “no EES” and “EES on” for each leg. Conditions with “no EES” and “EES on” demonstrated the applied force duration did not exceed the stance phase for both legs. Trials 1 and 2 during month 12 with “EES on” were not included because force values were deemed undeterminable. During month 18 (“no EES”), the applied force duration was similar to the stance phase, which indicates the clinicians had to provide assistance for the whole duration of stance. This was different for both legs during “EES on.” The duration of assistance decreased for the left leg where the overall applied force duration ranged from 0 to 2.15 s (Table 2B). This reduction in duration of applied force indicates progression toward independence on the left leg. However, the applied force duration for the right leg had more variability and it required more assistance for some trials.

## Discussions

Due to the nature of locomotor training being highly variable and relying heavily on hands-on cueing and support for individuals with SCI, it is plausible that the level of assistance during step training with EES can be related to the level of independence during stepping. During BWSTT, applied force was used to assist in stance phase of stepping to prevent knee buckling and to properly cue and engage the lower extremity extensor muscles. Two participants, both with sensorimotor complete SCI graded AIS-A, were able to improve independence of the stance phase in BWS treadmill stepping with optimal EES detected by FSRs positioned at the tibial tuberosity, which measured applied forces between clinicians' hands and participants' knees. During dynamic training with “no EES” and “EES on,” the FSRs were used to detect changes in force and duration of assistance during the stance or extension phase of the gait cycle to determine progression toward independence while using minimal BWS and slow speeds by month 18. The FSRs detected accurate point of contact and duration of assisted force at the knee related to increasing stance phase over the course of EES for both participants. Currently, there no clinical tools available to quantify assistance during BWSTT. The NeuroRecovery Scale assesses progression during step training in terms of independence from BWS, however, hands-on assistance is not graded (6). The Functional Independence Measure is a subjective scale used clinically and can grade overall assistance during overground walking, however, this is based on a relative percentage scale and is non-specific to phases of gait (15). Reduced assistance indicated during EES-enabled BWSTT for individuals with SCI is an important step forward in assessing optimization of stimulation and describing stepping progression. The FSRs were able to objectively note reduced clinician assistance and progression toward independence through quantifiable changes in applied force duration during the stance phase of the gait cycle.

The force was detected for most trials without a plateau except for participant 2 during month 12, where the FSR readings plateaued at ~80 N (Table 2A). During validation trials, where clinicians mimicked stance assistance on the ILL, the magnitude exceeded the active range resulting in a plateau compared to the gold standard at high levels of assistance noting a clear limitation. The assistance was detectable through the FSRs during BWSTT; however, the accuracy in magnitude was shown to be poor in validation testing, indicating an inability to quantify low, medium, and high forces directly from the FSR data reliably.

The FSR validation determined that we can accurately assess contact initiation and duration by the clinicians compared to the gold standard load cell. Reduced clinician assistance was evidenced by decreased durations of applied force on the FSRs at the knee during the use of EES. Participant 1 did not require hands-on application at the knee for consecutive steps bilaterally with the use of EES by month 18 (Figure 3). Participant 2 did not require hands-on application unilaterally with the use of EES by month 18 (Figure 6). This finding allowed us to indicate clinician assistance based on the duration of applied force as determined through FSRs relative to the stance phase.

Previously, Sayenko et al. reported FSR data from static standing demonstrating that external assistance at the knees decreased with increasing transcutaneous stimulation intensities, compared to no stimulation. Similar to observations in our study, the assistance during static activities was needed consistently during conditions with no stimulation (10). Previous subjective reports by our team regarding changes in assistance with the use of EES during BWSTT were not based on any quantitative scale but verbally reported post-testing (7). Additionally, Gill et al. report that lower BWS and intentional stepping resulted in greater independence and reduced clinician assistance. Quantifiable clinician assistance detected using FSRs during EES-enabled stepping can objectively describe performance changes. This technology could also be applied to other populations including those who have experienced stroke, traumatic brain injury, or those who have been diagnosed with multiple sclerosis to assess changes in clinician assistance in a variety of dynamic tasks including reaching, sit-to-standing activities, and overground walking. The FSRs could also be used to assess participant forces in assistive device applications. The data presented herein provide a method to quantify clinician assistance, which can identify progression toward independence based on the duration validation of FSRs. No practical, alternative tools exist to measure applied forces to assist a limb during EES-enabled BWSTT that longitudinally detect changes occurring during a highly dynamic and complicated task such as treadmill stepping.

Several strategies for improved force magnitude accuracy are apparent. One is to optimize the calibration procedure. All calibrations in this study were performed quasi-statically with the FSR laid flat on a hard surface; the environments in which the sensors are applied during participant assessments required them to be slightly curved and laid over a soft substrate. Additional experimentation to recreate the form, material interactions, and dynamics of the application environment may improve output. Another strategy may involve engineering the manner in which clinician forces are directed. The area of these FSRs is smaller than the trainer hands, and we cannot say with certainty that all forces are directed to the sensor and not shared with the surrounding tissue. A “handle” or some other fixture attached to the FSR may be engineered to better capture the transfer of force from the clinicians' hand to the sensor. Additionally, a sensor of a different, customized coverage area may be considered. Lastly, since we observed that the measurement range of these off-the-shelf sensors was not sufficient to capture the entire domain of forces for this activity, customizing the properties of a FSR to alter sensitivity and saturation load may be beneficial.

### Limitations

For the validation study, the 6-mm-thick Akton pad used may have had greater compliance than that of the soft tissue located at the knee on a human limb. A pad with lesser thickness may be more representative of tissue properties at the tibial tuberosity. The use of a single FSR between the two clinicians for the validation may have resulted in a degradation of quality of the sensor over time between testing bouts and limited the accuracy of grading low, medium, and high applied forces. Also, grading applied force simply as low, medium, and high may not create enough resolution to generate true differences. Application of this methodology during a participant's session with more trials, and where no EES, suboptimal EES, and optimal EES are used, may provide more concrete descriptions of assistance levels. Finally, the FSRs each have their own inherent range making it difficult to detect true trends between data collection timepoints.

The FSRs applied to rigid surfaces produced accuracy in measurements of timing of assistance; however, there are limitations in surface placement of device and consistent device performance requires consistent placement of hands on the sensors by trained clinicians. The conditions of use of the FSR in the clinical study were not strictly controlled as this was not a priority of the study, and this could have been a limitation on data analysis. Sensors composed of a more flexible material, which would mimics the skin texture, could be useful on less rigid surfaces allowing for movement with skin surface during dynamic activities. The FSRs were not applied to the medial hamstring for assessment of the swing phase. Alternate instrumentation is recommended for application to the medial hamstring and ankle region for swing evaluation. The shape and fixation method of the current FSRs would potentially impede the sensory cues necessary for the level of movement and variability in tissue density during swing phase. Force sensitive gloves could be an option; however, hands-on application of sensory cues during BWSTT are intended to be skin-to-skin contact. Further development of FSRs with a more pliable material may enable application to other locations where force is applied during step training, including the hamstrings and tibialis anterior and would enable a more complete picture of assistance throughout the gait cycle.

### Conclusions

Based on the results of this study, we conclude that FSRs could be applied to effectively measure clinicians' assistance and their corresponding applied force duration during dynamic rehabilitation combined with EES on and off. The FSR sensors effectively detected clinician assistance applied at the knees during dynamic rehabilitation with EES (both on and off) in terms of initial point of contact and duration of forces. However, accuracy of force magnitude was less impressive with this configuration. With this tool, we were able to observe reduced contact time by the clinicians that was related to increased stance duration, which objectively identified independence in stepping with the use of EES-enabled BWSTT following SCI. Future studies using FSRs to assess clinician assistance including other landmarks may provide a more complete picture of overall assistance during dynamic tasks. The FSRs have been demonstrated to provide accurate feedback on frequency and duration of clinician contact. With modifications to calibration techniques and custom-design strategies, absolute measures of force magnitude may be used detect clinician assistance and indicate progression during a variety of dynamic therapeutic interventions.

## Data Availability Statement

The raw data supporting the conclusions of this article will be made available by the authors, without undue reservation.

## Ethics Statement

The studies involving human participants were reviewed and approved by Mayo Clinic Institutional Review Board. The participants provided their written informed consent to participate in this study.

## Author Contributions

KDZ, MLG, PJG, and DGS initiated the project. ART designed the validation experiment. ART, MBL, and MLG performed the validation experiment. MBL, MLG, DDV, and KJF performed the rehabilitation for participants. ART, PJG, and JSC contributed to participant data collection. MBL, ART, and CL drafted the manuscript as well as completed revisions, analysis, and interpretation of data with subsequent contributions from all authors. MLG, DDV, RFH, JSC, DGS, and KJF assisted in revisions. KDZ supervised all aspects of this project and provided approval for the publication and content. All authors approved the final submission.

## Funding

KDZ received funding from the Jack Jablonski Bel13ve in Miracles Foundation, Mayo Clinic Rehabilitation Medicine Research Center, Mayo Clinic Transform the Practice, and Craig H. Neilsen Foundation. PJG was supported by Regenerative Medicine Minnesota and the Mayo Clinic Center for Regenerative Medicine. JSC was supported by the Mayo Clinic Graduate School of Biomedical Sciences.

## Conflict of Interest

The authors declare that the research was conducted in the absence of any commercial or financial relationships that could be construed as a potential conflict of interest.

## Publisher's Note

All claims expressed in this article are solely those of the authors and do not necessarily represent those of their affiliated organizations, or those of the publisher, the editors and the reviewers. Any product that may be evaluated in this article, or claim that may be made by its manufacturer, is not guaranteed or endorsed by the publisher.
